# ^1^H and ^15^N NMR Analyses on Heparin, Heparan Sulfates and Related Monosaccharides Concerning the Chemical Exchange Regime of the *N*-Sulfo-Glucosamine Sulfamate Proton

**DOI:** 10.3390/ph9030058

**Published:** 2016-09-07

**Authors:** Vitor H. Pomin

**Affiliations:** 1 Program of Glycobiology, Institute of Medical Biochemistry Leopoldo de Meis, Federal University of Rio de Janeiro, Rio de Janeiro 21941-590, Brazil; pominvh@bioqmed.ufrj.br or vhpomin@gmail.com; Tel.: +55-21-3938-2939; Fax: +55-21-3938-2090; 2 University Hospital Clementino Fraga Filho, Federal University of Rio de Janeiro, Rio de Janeiro 21941-913, Brazil

**Keywords:** heparan sulfate, heparin, NMR, *N*-sulfo-glucosamine, sulfamate proton

## Abstract

Heparin and heparan sulfate are structurally related glycosaminoglycans (GAGs). Both GAGs present, although in different concentrations, *N*-sulfo-glucosamine (GlcNS) as one of their various composing units. The conditional fast exchange property of the GlcNS sulfamate proton in these GAGs has been pointed as the main barrier to its signal detection via NMR experiments, especially ^1^H-^15^N HSQC. Here, a series of NMR spectra is collected on heparin, heparan sulfate and related monosaccharides. The N-acetyl glucosamine-linked uronic acid types of these GAGs were properly assigned in the ^1^H-^15^N HSQC spectra. Dynamic nuclear polarization (DNP) was employed in order to facilitate 1D spectral acquisition of the sulfamate ^15^N signal of free GlcNS. Analyses on the multiplet pattern of scalar couplings of GlcNS ^15^N has helped to understand the chemical properties of the sulfamate proton in solution. The singlet peak observed for GlcNS happens due to fast chemical exchange of the GlcNS sulfamate proton in solution. Analyses on kinetics of alpha-beta anomeric mutarotation via ^1^H NMR spectra have been performed in GlcNS as well as other glucose-based monosaccharides. 1D ^1^H and 2D ^1^H-^15^N HSQC spectra recorded at low temperature for free GlcNS dissolved in a proton-rich solution showed signals from all exchangeable protons, including those belonging to the sulfamate group. This work suits well to the current grand celebration of one-century-anniversary of the discovery of heparin.

## 1. Introduction

**Figure 1:**
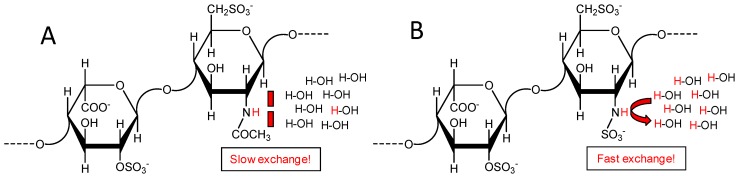
Structural representation of the main disaccharide building blocks of heparan sulfate [→4)-β-d-GlcA-(1→4)-α-d-GlcNAc-(1→] (**A**) and heparin [→4)-α-l-IdoA2S-(1→4)-α-d-GlcNS6S-(1→] (**B**). Although each unit can be seen in both GAG types, the first disaccharide is dominant in heparan sulfate while the second is more abundant in heparin. Structures highlight the different chemical exchange properties of the ^15^N-linked protons of the *N*-acetyl and *N*-sulfated groups with the protons from the bulk solvent. Analysis on the scalar coupling multiplet pattern related to direct observation of ^15^N in GlcNS is diagnostic of the chemical exchange regime of the amide proton.

Heparin and heparan sulfate are structurally correlated glycosaminoglycans (GAGs) endowed with multiple biomedical functions. They play key roles in coagulation, thrombosis, angiogenesis, cell proliferation, inflammation, microbial infections, tumor growth, metastasis, and many other pathophysiological systems [1,2,3]. Heparin and heparan sulfate are both composed of alternating 4-linked α-l-glucosamine (GlcN) and 4-linked uronic acid units within repeating disaccharide building blocks [2]. The uronic acid can be either β-d-glucuronic acid (GlcA), which is more abundant in heparan sulfate, or the C5 epimer α-l-iduronic acid (IdoA) which is dominant in heparin. In heparin, the IdoA unit is mostly 2-sulfated (IdoA2S). The GlcN units in both GAG types present, very often, additional *O*- and/or *N*-substitutions. The *O*-substitutions are the rare 3-*O*-sulfation and the common 6-*O*-sulfation. These *O*-sulfations occur more often in chains of heparin than of heparan sulfate. The *N*-substitutions are the *N*-acetylation (NHCOCH_3_) and the *N*-sulfation (NHSO_3_^−^) also known as sulfamate. These groups give rise to monosaccharides named respectively *N*-acetyl glucosamine (GlcNAc) and *N*-sulfo-glucosamine (GlcNS). While the former is dominant in heparan sulfate, the latter is more abundant in heparin [2]. The major repeating disaccharide units of heparan sulfate and heparin are respectively [→4)-β-d-GlcA-(1→4)-α-d-GlcNAc-(1→] (Figure 1A) and [→4)-α-l-IdoA2S-(1→4)-α-d-GlcNS6S-(1→] (Figure 1B).

Structural analyses on heparin and heparan sulfates are crucial, especially to establish further correlations with their biological roles. Nuclear magnetic resonance (NMR) spectroscopy is the most explored and informative analytical technique available so far for structural analyses of GAGs. Most of these analyses are based on the ^1^H and ^13^C nuclei. However, a great content of works using the less employed isotope ^15^N have been appearing in the literature lately [4,5,6,7,8,9,10,11,12,13,14,15,16,17,18,19,20,21,22,23,24]. This nucleus can be found at the composing amino sugars of GAGs such as GlcN, GlcNAc, GlcNS and *N*-acetyl galactosamine. The lower exploration of the ^15^N in NMR analyses of GAGs, as compared to the two other magnetic active nuclei, relies mostly on the lower sensitive of ^15^N. Recent developments such as the advent and spread of the use of ultrahigh magnetic fields, cryoprobe technology, isotopic labeling techniques, novel combinations of 2D pulse sequences and other modern techniques such as dynamic nuclear polarization (DNP) have, however, been facilitating the assessment of ^15^N-related resonances in NMR analyses of GAGs [4].

Here, a series of NMR-based experiments, mostly those involving ^15^N, has been applied to heparin, heparan sulfates and related monosaccharides, mostly GlcNS. It has been demonstrated that the uronic acid type (GlcA or IdoA) linked to the observable amino sugars of heparin and heparan sulfates can be properly identified via ^1^H-^15^N heteronuclear single quantum coherence (HSQC) spectra. Further NMR analyses, especially via 1D ^15^N signal acquisition assisted by DNP on the standard GlcNS monosaccharide, employed here as a model compound to the correspondent unit in heparin and heparan sulfate, have greatly supported the phenomenon of fast chemical exchange of the GlcNS sulfamate proton in solution, although the regime of the chemical exchange is highly conditional to other parameters such as pH, temperature and Ka. Analysis of the scalar coupling multiplet pattern of the 1D ^15^N in GlcNS will help to understand the phenomenon of fast exchange of the sulfamate proton in solution. Although other works exist in the field regarding the chemical exchange of the sulfamate proton, 1D ^15^N NMR for the investigation of this phenomenon has never been used befor. 1D ^1^H NMR experiments were here explored to (1) understand kinetics of anomeric mutarotation of some related monosaccharides, including GlcNS; (2) confirm the fast exchange property of the GlcNS sulfamate proton at regular experimental conditions; and (3) demonstrate the possibility of signal detection of this sulfamate proton when spectrum of GlcNS dissolved in a proton-rich solution is recorded at low temperature. This NMR-based study suits well to the current celebration of the 100th anniversary of the discovery of heparin.

## 2. Results and Discussion

### 2.1. Recognition of GlcNAc-Linked Uronic Acid Types in Heparin and Heparan Sulfates via ^1^H-^15^N HSQC Spectra

**Figure 2:**
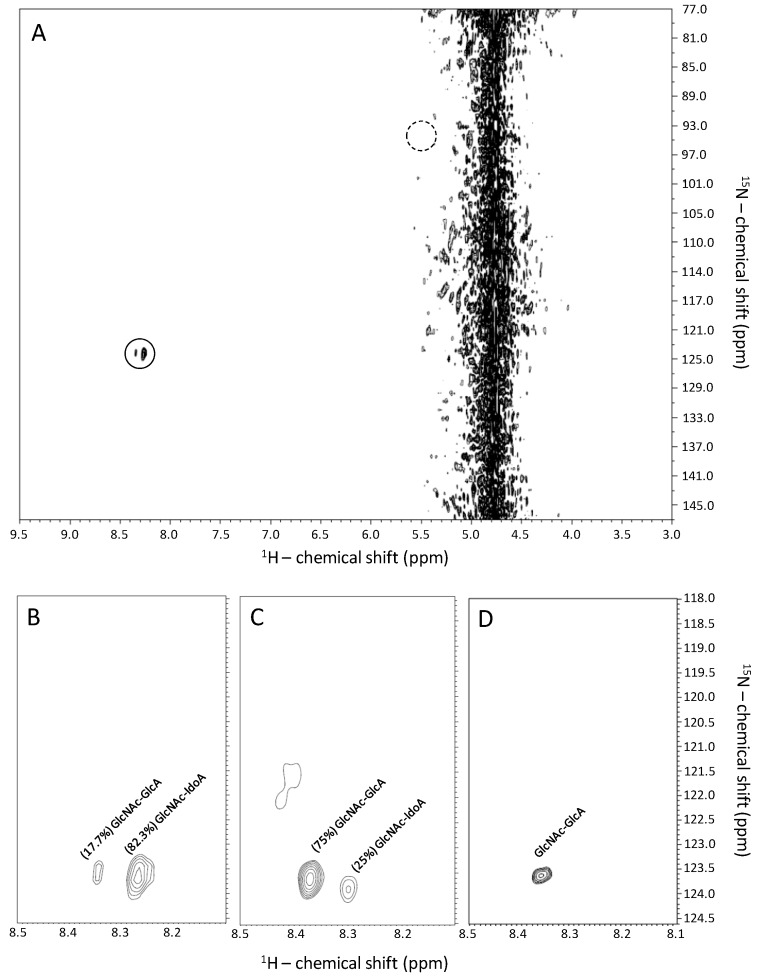
2D NMR ^1^H-^15^N HSQC spectra of heparin (**A**,**B**) and heparan sulfates isolated from Chinese Hamster Ovarian cells (**C**) and from the bivalve *Nodipecten nodosus* (**D**). Expansions are δ_H_/δ_N_ 3.0–9.5/77.0–147.0 ppm (**A**) and δ_H_/δ_N_ 8.1–8.5/118.0–124.6 ppm (**B**–**D**) for samples (10 mg/mL) dissolved in 50 mM sodium acetate buffer, 12.5% D_2_O (pH 4.5), 0.1% sodium azide. The solid and dashed circles highlight the observed and theoretical regions for the ^1^H-^15^N cross-peaks of *N*-acetyl glucosamine (GlcNAc) and *N*-sulfo-glucosamine (GlcNS) units, respectively. Spectra were recorded at 18.8 T and 25 °C.

The only two cross-peaks seen in a ^1^H-^15^N HSQC spectrum of a heparin-based sample dissolved in 50 mM sodium acetate buffer 12.5% D_2_O (pH 4.5) 0.1% sodium azide (final concentration of ~10 mg/mL) are highlighted in Figure 2A by a continuous-line circle. These cross-peaks resonating with δ_H_/δ_N_ at 8.32–8.37/123.4–123.8 and at 8.24–8.29/123.2–124.1 ppm belong to the amide group of GlcNAc units as assigned in previous works [4,5,6]. The reason for the appearance of these two resonances with different intensities has not been explained before. Relative abundance of these peaks has shown a proportion of 82%:18% (Figure 2B). The difference of percentages for the pair of resonances distinguished solely on the ^1^H dimension can be explained in terms of the composition of the adjacent uronic acid types linked to the GlcNAc units.

Heparin is well-known for bearing ≥70% IdoA units and the counter-balance of GlcA units [25]. The two GlcNAc-related ^1^H-^15^N cross-peaks result, therefore, from the GlcA/IdoA content in heparin structure. To support this interpretation and the assignments of the ^1^H-^15^N pairs in heparin as IdoA-linked and GlcA-linked GlcNAcs (Figure 2A,B); ^1^H-^15^N HSQC spectra of two heparan sulfates structurally distinct in terms of IdoA/GlcA ratios were included in the analyses (Figure 2C,D). The structures of these heparan sulfates were characterized in previous works [5,26]. The heparan sulfates were extracted from Chinese Hamster Ovarian (CHO) cells [5] and from the bivalve *Nodipecten nodosus* [26]. The IdoA percentage in mammalian-derived heparan sulfates has commonly a range of 30%–50% regardless of the origins and pathophysiological conditions [25]. The invertebrate heparan sulfate shows, on the other hand, a unique structure composed of chains entirely constituted of GlcA units [26]. The assignments of the ^1^H-^15^N cross-peaks with distinct percentages in the ^15^N-HSQC spectra of the two structurally distinct heparan sulfate samples can be associated with the differential GlcA/IdoA ratios reported to these two samples in the previous works [5,26]. As a consequence, the cross-peaks observed in the ^1^H-^15^N spectra for GlcNAc of heparin and heparan sulfates (Figure 2B–D) distinguished solely in terms of ^1^H-chemical shifts were attributed and assigned to a GlcA-linked GlcNAc NH resonance with downfield δ_H_, and to an IdoA-linked GlcNAc resonance with upfield δ_H_.

### 2.2. Fast Exchange Nature of the Sulfamate Proton in GlcNS from Heparin and Heparan Makes Difficult Its Signal Observation through ^1^H-^15^N HSQC Spectra at Regular Experimental Conditions

The cross-peaks observed in the ^1^H-^15^N HSQC spectra of heparin and heparan sulfates were assigned and attributed to the GlcNAc units (Figure 2). However, based on the background regarding the structure of these GAG species, GlcNAc (Figure 1A) is not the only the GlcN type present in their chains (Figure 1). The *N*-sulfated GlcN unit (Figure 1B), usually represented as GlcNHSO_3_^−^ or GlcNS, is also present, and the GlcNAc/GlcNS ratio varies significantly among heparin and heparan sulfates (Figure 1A vs. Figure 1B). Based on the knowledge regarding the mammalian-derived GAGs, it has been generally accepted that while GlcNS unit occurs in heparin within a concentration of ≥80%, in heparan sulfates it happens in a range of 40%–60% [25]. Despite the significant concentrations of GlcNS units in both GAG types, ^1^H-^15^N cross-peaks derived from this unit have not been detected through the series of ^1^H-^15^N HSQC spectra depicted at Figure 2. Many previous works have, however, observed and identified the ^1^H-^15^N cross-peak of the GlcNS unit [7,16,18,19,20,23,24]. The ^1^H-^15^N pair of GlcNS resonates to a far upfield region than GlcNAc in both ^1^H and ^15^N dimensions, more exactly close to δ_H_/δ_N_ at 5.5/93.5 ppm [16]. This region has been highlighted in the ^1^H-^15^N HSQC spectrum of Figure 2A by a dashed-line circle. The NH signal of GlcNS can be detected at natural abundance by choosing the proper pH condition in which the exchange with the bulk solvent can be reduced [7].

### 2.3. Non-polarized and Hyperpolarized 1D ^15^N and ^1^H Direct-observe of ^15^N-Gln, GlcNS and Other Monosaccharides

**Figure 3:**
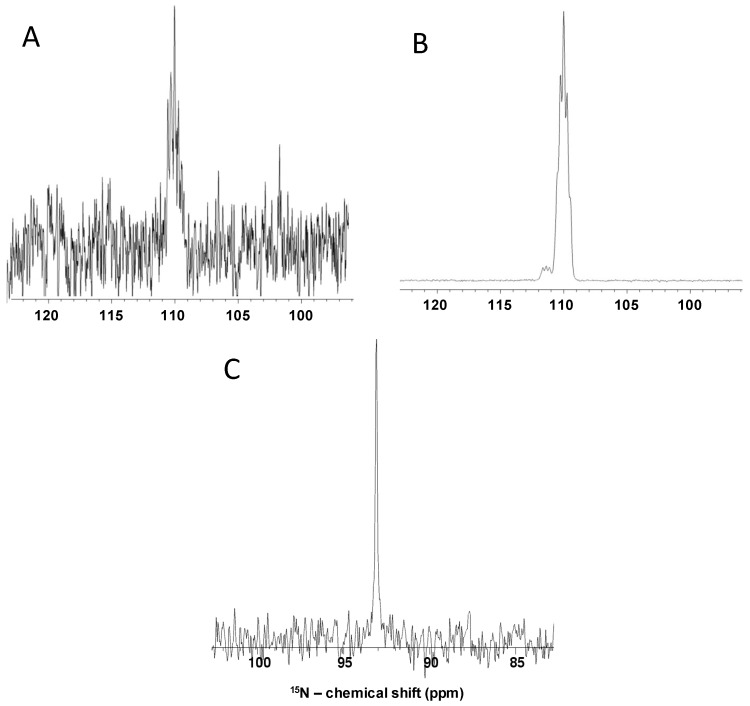
Comparison of the 1D NMR spectra ^15^N direct-observe of the ^15^N-isotopically labeled side chain glutamine (^15^N-Gln) at 23 mM with 98% ^15^N abundance (**A**,**B**) versus *N*-sulfo-glucosamine (GlcNS) at 20 mM with ^15^N natural abundance (0.37%) (**C**) at non-polarized (**A**) and hyperpolarized conditions (**B**,**C**). All spectra were recorded at 11.7 T and 37 °C.

Figure 3 displays 1D ^15^N signals for ^15^N-labeled side chain Gln (panels A and B) and GlcNS at isotopic natural abundance (panel C) acquired at non-polarized (panel A) and hyperpolarized (panels B and C) conditions. The ^15^N signals of ^15^N-Gln (Figure 3A,B) and GlcNS (Figure 3C) resonate with δ_N_ at 109.93 and at 93.1 ppm, respectively. Theoretical calculations lead to a factor of four-thousand-fold enhancement from the non-polarized (Figure 3A) to the hyperpolarized (Figure 3B) condition. A time course of one month-long acquisition in average would be necessary to record a 1D ^15^N spectrum with same signal-to-noise ratio of the hyperpolarized ^15^N-Gln (Figure 3B) via a non-polarized experiment (Figure 3A) on the same magnetic field (500 MHz). This indicated that sensitivity of ^15^N direct-observe through the non-polarized method, even on a ^15^N-labeled molecule of low-molecular weight such as ^15^N-Gln, can be very difficult. To achieve the optimal sensitivity, the experiment would be unreasonably time-consuming. On the other hand, resolution can be enhanced in a faster way for both labeled (Figure 3B) and unlabeled compounds (Figure 3C) via the hyperpolarization technique. ^15^N-Gln was used initially here for setting up the experimental parameters and to understand the potentiality of the DNP in enhancing sensitivity for spectral acquisition. This standard turned out to be useful as a tool in comparative analyses of the multiplicity patterns of the ^15^N peak of GlcNS (Figure 3C) as compared to ^15^N-Gln (Figure 3B).

The spectrum from the ^15^N direct-observe of ^15^N-Gln displays a resonance with splitting characterized by a triplet pattern accompanied by a small triplet signal on left due to isotopic shift (Figure 3B). The triplet pattern occurs due to the presence of two amide protons coupled to ^15^N. On the other hand, the GlcNS produces just a single singlet peak with the ^15^N-direct observation (Figure 3C). This singlet pattern occurs due to fast exchange of the sulfamate proton with the solvent under the conditions of the experiment. The exchange property of the amide protons in Gln as compared to the one from GlcNS is much slower due to the absence of a nearby electronegative chemical group like sulfation.

### 2.4. Kinetic of the α-β Mutarotation Monitored through Anomeric ^1^H Resonances

**Figure 4:**
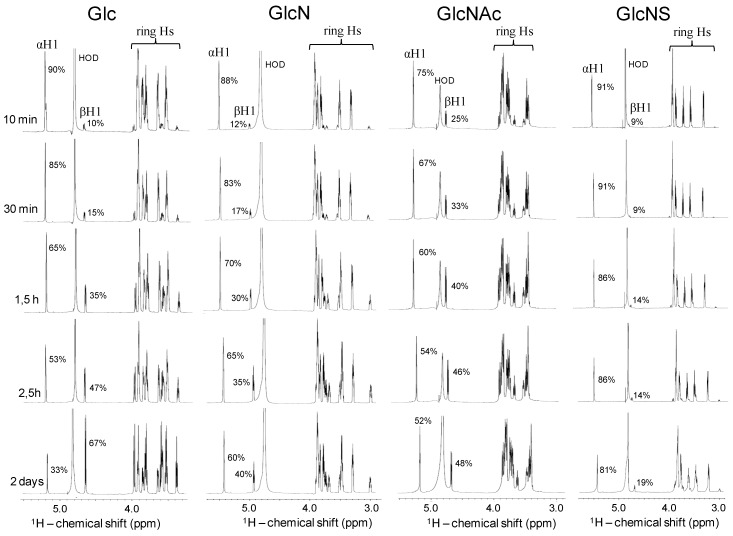
Kinetics of anomeric mutarotation observed for glucose (Glc) and Glc-based standards such as glucosamine (GlcN), *N*-acetyl glucosamine (GlcNAc) and *N*-sulfo-glucosamine (GlcNS) measured through 1D ^1^H NMR spectra recorded within different time courses after dissolution in 100% D_2_O (10 mg/mL). Anomeric proton signals (αH1 and βH1), their respective relative percentages and ring proton signals are indicated in the panels. HOD denotes residual water signals. Spectra were recorded at 18.8 T and 25 °C.

After questioning about the chemical properties of the sulfamate proton of the GlcNS units (either as composing unit in the backbones of GAGs or as free monosaccharide in solution) and analyzing the DNP-assisted 1D ^15^N signal pattern of this monosaccharide, questions about the anomeric mutarotation kinetics of this unit compared to other related and commoner monosaccharides also raised. To address this particular point kinetics of the α↔β anomers of GlcNS was further investigated comparatively with other Glc-based standards, via the commoner and faster 1D ^1^H NMR method (Figure 4). As opposed to the three first standards (Glc, GlcN and GlcNAC) that showed faster kinetics in the first hours after sample dissolution, the α:β ratio of GlcNS changes, interestingly, just slightly from the initial great dominance of the α-form (90% at 10 min) to the point which the equilibrium is reached (80:20 for the α:β ratio after 2 days dissolution). As seen, the GlcNS presents a major anomeric conformation in solution which is the α-form (Figure 4). These monosaccharide standards were used in the investigation rather than heparin-derived disaccharides because of their commercial availability and reduced structural variations.

### 2.5. Reducing the Fast Exchange Rates of the GlcNHSO_3_^−^ to Enable Proper NMR Detection

Since the proton of the sulfamate group in GlcNS free in solution or as composing units of heparin/heparan sulfate chains is not easily observed through NMR spectra (Figure 3C and Figure 2A) recorded respectively at room (25 °C) and physiological temperatures (37 °C), the development of an experimental condition for detection of this particular proton through NMR spectroscopy seems valuable. This condition relies on dissolving the GlcNS standard in a mixture of 10%:20%:70% D_2_O/acetone/H_2_O solution and then to record NMR spectra at 3 °C. The low temperature helps to slow down the rapid chemical exchange of the labile protons during the NMR experiment. The residual D_2_O in the solution was used for deuterium-lock in the instrument, acetone was used for avoiding freezing of the sample at low temperature and abundance of H_2_O was explored to force protonation.

**Figure 5:**
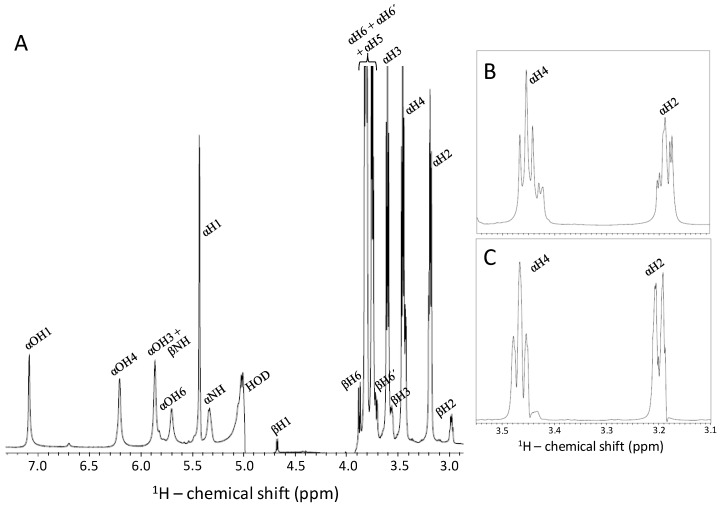
1D ^1^H NMR spectra of *N*-sulfo-glucosamine (GlcNS). Expansions are 2.9–7.3 ppm (**A**) and 3.10–3.55 ppm (**B**,**C**) for GlcNS (10 mg/mL) dissolved in 10%:20%:70% D_2_O/acetone/H_2_O for spectra recorded in 18.8 T NMR instrument at 3 °C (**A**,**B**) or 37 °C (**C**). Spectrum in panel B is just a close-up window of the correspondent region in spectrum of panel A.

The 1D NMR spectrum of the GlcNS sample obtained after following this experimental strategy is displayed in Figure 5A. This spectrum demonstrates that not only the sulfamate proton resonance, but in fact, all exchangeable protons of GlcNS have become accessible. Signals of the exchangeable protons (nitrogen- or oxygen-linked protons) were rather less sharp than the unexchangeable ones. The line-broadening and low intensity of the exchangeable proton resonances arise from a residual effect from the continued chemical exchange and dynamics, even at reduced temperature. Nonetheless, peaks were well-resolved and intensities were sufficient for chemical shift measurements and to attempt further 2D spectral acquisition on the GlcNS. Although the detection and assignment of the exchangeable protons of GlcNS have been reported in previous works [8,22], the current 1D ^1^H NMR data present here is useful to demonstrate the differential multiplicities of the αH2 resonance with δ_H_ at ~3.2 ppm upon low and fast proton exchange regime (Figure 5B,C) as discussed further.

Assignments of all resonances in this 1D spectrum (Figure 5) were accomplished by tracing spin-spin connectivities (spin systems) in the 2D TOCSY spectrum recorded also at 3 °C for the GlcNS sample (Figure S1A). In this TOCSY spectrum, the labile protons from hydroxyl and amide groups are accordingly labeled as OH and NH together with the unexchangeable protons ascribed as αH1, βH1, αH2, βH2, αH3 and αH4. Chemical shift values for these ^1^H resonances are plotted in Table S1. Acquisition of a ^13^C-HSQC spectrum of this GlcNS sample at 3° C temperature was also achieved (Figure S1B). Assignments of ^1^H-^13^C cross-peaks were based on the previously identified ^1^H-chemical shifts obtained via TOCSY spectrum. The chemical shifts for all ^13^C-atoms were subsequently obtained after the ^1^H-chemical shift assignments (Table S1).

### 2.6. Fast Chemical Exchange of GlcNHSO_3_^−^ and Validation of the Low-Temperature-and-Proton-Rich-Solution Condition for NMR Detection as Seen by ^1^H Coupling

Note that in panel B of Figure 5 there is a triplet of doublets on the resonance of αH2 of GlcNS (δ_H_ at 3.16–3.22 ppm). This is likely due to the splitting phenomenon caused by ^3^*J*_H2-H1_, ^3^*J*_H2-H3_ and ^3^*J*_H2-HN_ at 3 °C temperature. However, when the temperature is raised to the physiological one (Figure 5C), the multiplet profile of the αH2 resonance moves to a doublet of doublets (^3^*J*_H2-H1_ and ^3^*J*_H2-H3_ of same constant values) demonstrating thus that the ^3^*J*_H2-HN_ was lost due to enhanced dynamics and faster chemical exchange of the sulfamate proton. Although chemical shifts are highly sensitive to temperature [14], the comparative analyses on the multiplet patterns in the spectra recorded in this work at different temperatures (3 °C at Figure 5B and 37 °C at Figure 5C) have not compromised the interpretation and conclusions of the results.

After designing, testing and probing the experimental protocol for assessing exchangeable protons in GlcNS (Figure 5 and Figure S1), a ^15^N-HSQC spectrum of the GlcNS sample was recorded following the same strategy (Figure S2). The spectrum of Figure S2 is represented in two different thresholds because of the proximity of the upfield peak of the NH pair of α-GlcNS (δ_H_/δ_N_ at 5.36/93.9 ppm) with the broad residual water noise peak (δ_H_ ranging from 5.35 to 4.87 ppm). The ^1^H-^15^N cross-peak of the NH pair of β-GlcNS resonates more downfield in the spectrum, with δ_H_/δ_N_ exactly at 5.91/93.6 ppm. Percentage of these amide resonances relative to the α- and β-configurations of GlcNS was measured based on integral values of the ^1^H-^15^N cross-peaks. The values obtained are in total accordance with the expected α/β-anomeric ratio observed for GlcNS in equilibrium as measured previously by 1D ^1^H-NMR (Figure 4). Hence, the special protocol for decreasing rapid chemical exchange of the sulfamate protons in GlcNS and to enable signal detection via both 1D ^1^H (Figure 5A) and ^15^N-HSQC NMR spectra (Figure S2), both recorded at 3 °C after dissolving powder GlcNS in a mixture of 10:20:70: D_2_O:acetone:H_2_O solution, was probed to be functional.

## 3. Materials and Methods

### 3.1. General Materials

The sodium salt of heparin (unfractionated heparin) was gently provided by Eurofarma, Itapevi, Brasil. The 3 and 8 mm glass NMR tubes were purchased from Tedia, Rio de Janeiro, Brasil. ^15^N-labeled side chain glutamine (^15^N-Gln with ^15^N at 98%) and deuterium oxide “100%” (D99.96%) were purchased from Cambridge Isotope Laboratories, In. (Andover, MA, USA). The GlcNS (d-Glucosamine-2-*N*-sulfate sodium salt) was purchased from Carbosynth (Berkshire, UK). The heparan sulfate from CHO K1 cells and from the bivalve *Nodipecten nodosus* were the same utilized in the previous publications [5,26]. Glucose (Glc) (d-(+)-Glucose ≥99.5%), GlcN (d-(+)-Glucosamine hydrochloride ≥99%, crystalline) and GlcNAc (*N*-Acetyl-d-glucosamine ≥99%) and reagents for buffer preparation like sodium acetate, sodium azide, dibasic sodium phosphate, citric acid and acetone were purchased from Sigma Aldrich Co. (St. Louis, MO, USA).

### 3.2. NMR Experiments, Instrumentation and Related Reagents

For each hyperpolarization experiment, a solution of 23 mM ^15^N-Gln or 20 mM GlcNS was obtained dissolving gently the powder compounds in 25:25:50 (*v*/*v*/*v*) D_2_O:H_2_O:glycerol containing 15 mM trityl radical (GE-Healthcare, Buckinghamshire, UK) in heating (70 °C). This mixture was added to a polyether ether ketone plastic cup and lowered into an Oxford Hypersense 3.35 T DNP polarizer (Oxfordshire, UK). Samples were cooled to 1.4–1.5 °C then irradiated for 1–2 h at 94.007 GHz and 100 mW. The hyperpolarized samples were then quickly melted and dissolved in a buffer 25 mM citric acid, 25 mM dibasic sodium phosphate (pH previously adjusted to ~7.0). The dissolved material was automatically flushed into a waiting 8 mm NMR tube in a Varian 11.7 T Inova spectrometer (Santa Clara, CA, USA) equipped with an XH probe operating at a 37 °C sample temperature (∼5 s transfer time). Each sample was analyzed and experimentally performed separately.

To assess the kinetics of anomeric mutarotation of Glc-based standards, approximately 1.5 mg of the standard monosaccharides (dried weight) was dissolved in 160 μL 100% D_2_O and transferred into 3 mm NMR tubes for 1D ^1^H spectral acquisition. To assess exchangeable proton resonances in GlcNS, approximately 1.5 mg of powder GlcNS was dissolved in 160 μL 10:20:70% D_2_O:acetone:H_2_O (the pH after dissolution was measured as ~6.5) and transferred into a 3 mm NMR tube for 1D ^1^H spectral acquisition. For ^1^H-^1^H Total Correlation SpectroscopY (TOCSY), ^1^H-^15^N HSQC and ^1^H-^13^C HSQC spectral acquisition of samples (GlcNS, heparin or heparan sulfates) around 1.5 mg of dried weight material was dissolved in either 160 μL 10%:20%:70% D_2_O/acetone/H_2_O (the pH after dissolution was measured as ~6.5) or in 160 μL 50 mM sodium acetate buffer 12.5% D_2_O (pH 4.5) 0.1% sodium azide (as indicated in figure captions) and transferred into a 3 mm NMR tube.

All NMR experiments were recorded on Varian Inova spectrometer (Santa Clara, CA, USA), with a triple resonance cold probe operating at 800 MHz (18.8 T) or 500 MHz (11.7 T) for the ^1^H Lamor frequency. During NMR spectra collection, temperatures of 3 (protonated sulfamate), 25 or 37 °C (unprotonated sulfamate) were used as indicated in figure captions. The 1D ^1^H spectra were recorded with 128 scans with a spectral width of 7 kHz, carrier position at the HOD peak (4.8 ppm), acquisition time set to 2 s, and water presaturation pulse (when used) set to the position of the carrier for a period equal to the recovery delay (1.5 s). The 1D ^15^N-direct-observe spectra of ^15^N-Gln were recorded using the ^15^N channel with similar parameters used for 1D ^1^H except 90° pulse width for ^15^N and the lack of presaturation pulse.15k scans were used for 1D ^15^N direct-observe in the non-polarized sample. The ^1^H-^1^H TOCSY spectra were run with spectral widths of 6 kHz, and acquisition time of 175 ms using 96 scans per t1 increment (64 points) to achieve a time domain matrix of 2110 × 128 complex points, using a spin-lock field of 9 kHz, and a mixing time of 60 ms. ^1^H-^13^C HMQC spectra were run with acquisition time of 0.128s using 144 scans per t1 increment (128 points) to achieve a time domain matrix of 1366 × 256 complex points. ^1^H-^15^N HSQC spectra were recorded with acquisition time of 0.095s and with192 scans per t1 increment (128 points) to achieve a time domain matrix of 1366 × 256 complex points. All processing was done with NMRPipe software [27]. All spectra were apodized in both dimensions with the automatic function cosine-bells, together with automatic zero-filling to double the sizes followed by rounding to the nearest power of 2. Reported chemical shifts for ^1^H, ^13^C and ^15^N are relative to the trimethylsilylpropionic acid, methanol and liquid ammonia, respectively.

## 4. Conclusions

In this approach, a series of NMR spectra have been collected for heparin, heparan sulfates and related monosaccharides. The standard GlcNS monosaccharide was the most analyzed sample, especially via methods involving the less used and less sensitive ^15^N isotope. The standard GlcNS monosaccharide was used here as a model compound to the composing GlcNS unit in heparin and heparan sulfate. DNP was employed to facilitate 1D signal acquisition of ^15^N, and spectra recorded at low temperature after dissolution of the samples in a proton-rich solution were studied. Data obtained from this current NMR-based study have strongly supported the conception that the sulfatame proton in free standard GlcNS monosaccharide (Figure 3C and Figure 5C) as well as composing GlcNS units of the heparin and heparan sulfate chains, analyzed at either room (Figure 2) or physiological temperatures (Figure 5C), occurs in fast chemical exchange in solution under the pH achieved here by just dissolving the sample in the used solvents. The rapid chemical exchange of the sulfamate proton is pH-dependent and choosing the correct pH range for work, detection of the GlcNS NH is possible [7]. Besides pH, temperature is another contributing factor to the slow-fast exchange regimes of the sulfamate proton as well as for the other exchangeable protons. This work has shown a strategy based on low temperature to reduce the fast chemical exchange property of GlcNHSO_3_^−^ to a point where proper signal detection can also be achieved by NMR. The current investigation on this key structural element of heparin is of great relevance in light of the ongoing grand celebration of one-hundred-year-anniversary of the discovery of heparin. As known, GlcNS is a functional unit in chains of heparin and its sister GAG heparan sulfate, especially inside biding sequences responsible for interactions with antithrombin and growth factors during events of anticoagulation and cell division. Heparin is the most therapeutic carbohydrate, largely employed as anticoagulant in the clinic. The structural studies on the biologically active structural elements of this GAG type are therefore valuable.

## Supplementary Materials

The following are available online at http://www.mdpi.com/1424-8247/9/3/58/s1, Figure S1: 2D NMR ^1^H-^1^H TOCSY (A) and ^1^H-^13^C HSQC (B) spectra of *N*-sulfo-glucosamine (GlcNS) (10 mg/mL) dissolved in 10%:20%:70% D_2_O/acetone/H_2_O, recorded at 18.8 T and 3 °C (A); Figure S2: 2D NMR ^1^H-^15^N HSQC spectrum of GlcNS (5 mg/mL) dissolved in 10%:20%:70% D_2_O/acetone/H_2_O recorded at 18.8 T and 3 °C displayed at higher (A) and lower (B) counter levels; Table S1: Chemical shifts of carbon-attached unexchangeable ^1^H from both α and β-anomeric configurations, oxygen-linked exchangeable ^1^H from α-anomeric configuration, nitrogen-linked exchangeable ^1^H from α- and β-anomeric configurations, and ^13^C of α-anomeric configuration of GlcNS as assigned in spectra of Figures S1A, S2A and S2B.
