# Supplementary material for: 1H and 15N NMR Analyses on Heparin, Heparan Sulfates and Related Monosaccharides Concerning the Chemical Exchange Regime of the N-Sulfo-Glucosamine Sulfamate Proton
Source: Pharmaceuticals (Basel). 2016 Sep 7;9(3):58. doi: 10.3390/ph9030058 (PMC5039511; doi:10.3390/ph9030058)
Supplement: Supplementary file 1 [file pharmaceuticals-09-00058-s001.pdf]

Supplementary Materials: ^{1}H and ^{15}N NMR Analyses on Heparin, Heparan Sulfates and Related Monosaccharides Concerning the Chemical Exchange Regime of the *N*-Sulfo-Glucosamine Sulfamate Proton

Vitor H. Pomin

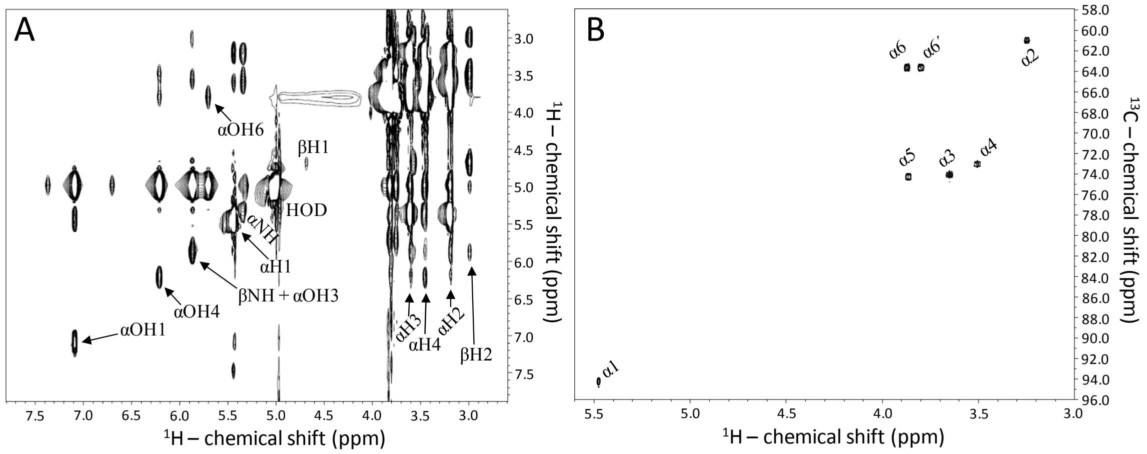

Figure S1. 2D NMR ^{1}H-^{1}H TOCSY (**A**) and ^{1}H-^{13}C HSQC (**B**) spectra of *N*-sulfo-glucosamine (GlcNS) (10 mg/mL) dissolved in 10%:20%:70% D_2O/acetone/H_2O, recorded at 18.8 T and 3 °C (**A**).

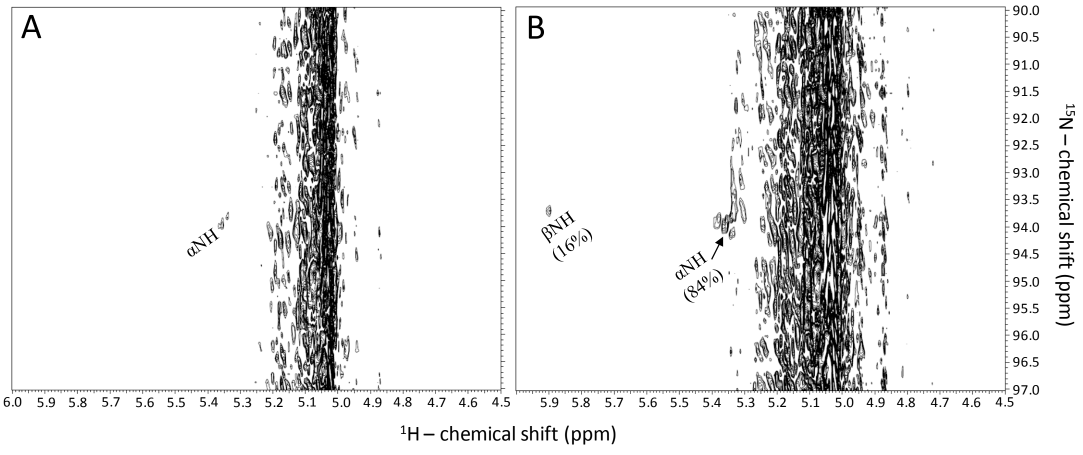

Figure S2. 2D NMR ^{1}H-^{15}N HSQC spectrum of GlcNS (5 mg/mL) dissolved in 10%:20%:70% D_2O/acetone/H_2O recorded at 18.8 T and 3 °C displayed at higher (**A**) and lower (**B**) counter levels.

Table S1. Chemical shifts of carbon-attached unexchangeable ^{1}H from both α and β-anomeric configurations, oxygen-linked exchangeable ^{1}H from α-anomeric configuration, nitrogen-linked exchangeable ^{1}H from α- and β-anomeric configurations, and ^{13}C of α-anomeric configuration of GlcNS as assigned in spectra of Figures S1A, S2A and S2B.

^{1}H and ^{13}C Chemical Shifts (ppm) [a]			
αH1	5.44	αOH1	7.09
αH2	3.19	αOH3	5.86
αH3	3.60	αOH4	6.20
αH4	3.43	αOH6	5.70
αH5	3.82	αNH	5.32
αH6	3.81	βNH	5.86
αH6′	3.54	αC1 [c]	94.2
βH1	4.68	αC2	61.0
βH2	2.99	αC3	74.0
βH3	3.57	αC4	73.0
βH4	Nd [b]	αC5	74.3
βH5	nd	αC6	63.6
βH6	3.89	αC6′	63.6
βH6′	3.72	-	-

[a] ^{1}H and ^{13}C chemical shifts are relative to the trimethylsilylpropionic acid and methanol respectively. [b] not determined. [c] ^{13}C-chemical shifts were plotted in table due to the greater resolution of the α-configuration of GlcNS in spectrum of Fig. S1B, although chemical shifts of all ^{13}C atoms of the β-configuration were also safely determined (βC1 at 95.9, βC2 at 59.9, βC3 at 75.2, βC4 at 73.0, βC5 at 72.5, βC6 and βC6′ at 93.7 ppm).

© 2016 by the authors; licensee MDPI, Basel, Switzerland. This article is an open access article distributed under the terms and conditions of the Creative Commons by Attribution (CC-BY) license (http://creativecommons.org/licenses/by/4.0/).
